# Emerging role of radiation induced bystander effects: Cell communications and carcinogenesis

**DOI:** 10.1186/2041-9414-1-13

**Published:** 2010-09-12

**Authors:** Rajamanickam Baskar

**Affiliations:** 1 Department of Radiation Oncology, Division of Cellular and Molecular Research, National Cancer Centre, Singapore

## Abstract

Ionizing radiation is an invaluable diagnostic and treatment tool used in various clinical applications. On the other hand, radiation is a known cytotoxic with a potential DNA damaging and carcinogenic effects. However, the biological effects of low and high linear energy transfer (LET) radiations are considerably more complex than previously thought. In the past decade, evidence has mounted for a novel biological phenomenon termed as "bystander effect" (BE), wherein directly irradiated cells transmit damaging signals to non-irradiated cells thereby inducing a response similar to that of irradiated cells. BE can also be induced in various cells irrespective of the type of radiation, and the BE may be more damaging in the longer term than direct radiation exposure. BE is mediated either through gap-junctions or via soluble factors released by irradiated cells. DNA damage response mechanisms represent a vital line of defense against exogenous and endogenous damage caused by radiation and promote two distinct outcomes: survival and the maintenance of genomic stability. The latter is critical for cancer avoidance. Therefore, efforts to understand and modulate the bystander responses will provide new approaches to cancer therapy and prevention. This review overviews the emerging role of BE of low and high LET radiations on the genomic instability of bystander cells and its possible implications for carcinogenesis.

## Introduction

Extensive epidemiological and toxicological research over several decades has focused on the health effects of radiation to understand the risk of exposure to both public and workforce. Ionizing radiation has been used in both diagnostic and therapeutic medical applications and described as a double-edged sword [1]. However, there are considerable concerns about the detrimental health effects associated with direct radiation exposure [1-3] even on metabolically inactive cells [4,5]. Radiation is harmful in terms of risks to health from accidental exposure and its role as a carcinogen [6], however on the other side it is beneficial for the use of various diagnostic and therapeutic procedures such as the treatment of cancer. Radiotherapy (RT) continues to be an important therapeutic modality for the treatment of cancer. RT for cancers allows killing of the cancer cells but also shows a risk for adverse consequences such as tissue atrophy and formation of secondary tumors at the same organ, or at some distanced part of body [7]. Furthermore, radiation exposure during diagnostic (e.g. X-rays, CT-scans) and RT procedures shows varying health effects in the general population and also in cancer patients [8-14]. But with cancer survivors living longer, there is a growing concern regarding the risk of radiation**-**induced secondary cancers in patients treated with ionizing radiation. The situation is important for children, who are inherently more radiosensitive and therefore at greater risk for radiation induced post-radiotherapy cancer development [15-17].

**Figure 1:**
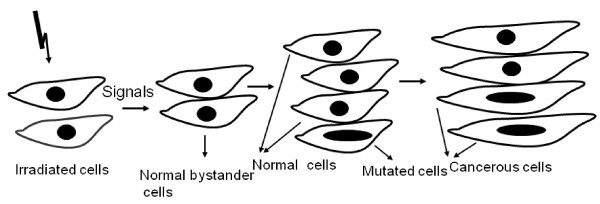
**Schematic representation of signals in the form of soluble factors released from irradiated cells to distanced non-irradiated (bystander) cells**. Damage caused in bystander cells in the form of mutation may lead to cancer formation.

**Figure 2:**
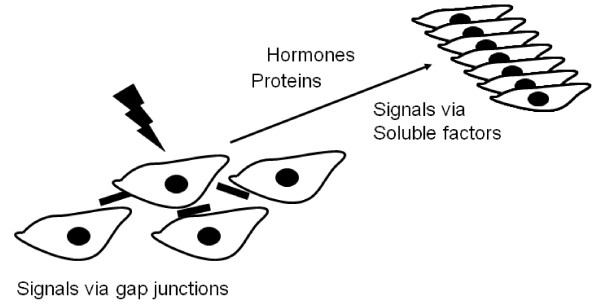
**Schematic representation of signals released from the irradiated cells passes through gap junctions to nearby (adjacent) cells and soluble factors (proteins and hormones) to distanced cells/organs**.

**Figure 3:**
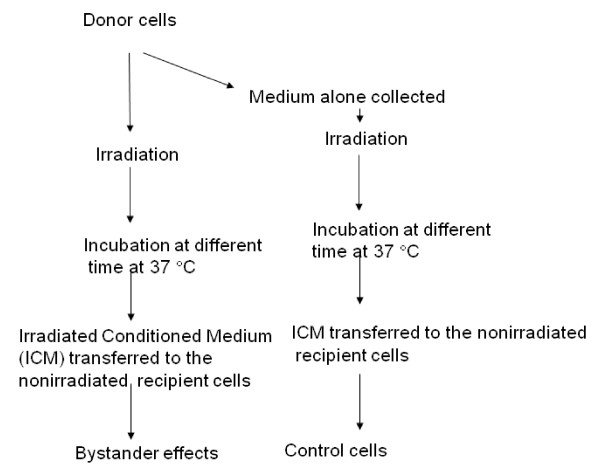
**Schematic representation of procedures followed for medium transfer treatment techniques from irradiated to the non-irradiated (bystander) cells**.

The DNA damage response system, which maintains the survival and genomic stability of the cell, represents a vital line of defense against various exogenous and endogenous DNA damaging agents [18]. Radiation can induce apoptosis or trigger DNA repair mechanisms. In general minor DNA damage is thought to halt cell cycle to allow effective repair, while more severe damage can induce an apoptotic cell death program. Until relatively recently, the only known adverse consequences of radiation exposure to the cells were direct DNA damage. Damage of DNA is central to major biological processes such as cancer, disturbances of cell function and aging [19,20]. Particularly, genome stability is critical for the avoidance of cancer development. In normal cells, DNA damage of a single cell most likely represents an initiating event for carcinogenesis. However, the dogma that genetic and biochemical alterations are restricted only to the directly irradiated cells has been challenged by the observation that similar effects can also be seen in normal non-irradiated cells adjacent to the irradiated or targeted cells [4,6,21-26], known as "bystander effect" (BE). The consequence of this transmitted signal could be either negative or positive, depending on the specific circumstances. BE results from the ability of cells directly irradiated to produce changes in normal cells nearby, which are not directly irradiated, thereby eliciting similar responses to that of the irradiated cells (Figure-1). The transmission of signal(s) appears to be via secreted soluble molecules [23,27,28] or via gap junctions [29-31] (Figure-2). Most BE studies have been carried out using various cell lines (*in vitro*), where irradiated medium is transferred to non-irradiated cell (Figure 3). In this review, I have summarized the current understanding of radiation induced BE, with a focus on cell communications and its implications towards genomic instability.

## Type of radiations and bystander effect

Biological effectiveness of radiation depends on the linear energy transfer (LET), total dose, fractionation rate and radio-sensitivity of the targeted cells or tissues [10]. Low LET radiations (X-rays, gamma rays and beta particles), deposi**s**ts a relatively small quantity of energy. On the other hand, high LET radiations (neutrons and alpha particles) deposits more energy on the targeted areas and caused more biological effects than the low LET radiations. Although various precautions are taken during RT to limit the damage to healthy normal tissues surrounding the target site, potential injury can lead to various normal-tissue complications. In addition to direct effects, bystander effects also show dependency on the type of radiation and appear to be cell type and genotype specific [23]. This suggests a need to understand the mechanism(s) for radiation induced bystander effects.

The direct and indirect (bystander) radiation effects are mechanistically different. It has been shown that the intensity of damage and radiation injury varies according to the specific cell type and its susceptibility to the radiation injury. For example, radiotherapy of pelvic cancer is frequently associated with the early and late intestinal and rectal toxicity [32]. Both low and high LET radiations have been shown to induce BE [4,23,29,32-37]. However, it is not clear whether the BE is confined to both type of radiation exposure of various systems at different end points studied. Studies on the BE carried out falls into two main approaches: 1. Use of low and high LET radiations (whole cells irradiated); 2. Targeting the cellular organelles using an advanced microbeam radiation facilities, this is available only in few countries.

In the whole cell irradiation experiments either after low or high LET radiations, transferring irradiated conditioned medium (ICM) from the irradiated cells to the non-irradiated bystander cells were used. Many signal molecules remain bound to the surface of the irradiated cell and influence only cells that contact it, or these signals can be soluble in its environment, which can be carried far from the irradiated cells. Contact-dependent signaling is important only in nearby cells or within organs. In most cases, paracrine or endocrine signaling molecules are carrying the secreted stimuli by the irradiated cells to act as a local or far a field. The signaling molecules can be gases, proteins or hormones. For example nitric oxide (NO), a gas and part of a locally based signaling system is able to lower human's blood pressure is indicated in the radiation induced bystander responses [23,38,39]. Thus, the mechanical injury caused by a direct irradiation can activate various chemical stimuli and communicate to nearby or far located cells that have not been directly influenced by irradiation. Molecules secreted in response to radiation may also be carried far from the secreted cells to the distant targets, or they may act as local mediators only affecting cells in the immediate environment of the signaling cell (Figure-2). Another way to coordinate the activities of neighboring cells is through gap junctions. Cell-cell junctions can form through the plasma membranes closely adjacent cells, connecting their cytoplasm. This type of communication system operates on a local level. Although gap junction communication and the presence of soluble mediator(s) are both known to play an important role in bystander response, the precise signaling molecules have yet to be identified. What is not fully understood at this juncture is how the damaged cell passes its damage signals to the normal cells located at far distance. One possible mechanism is via hormones. Hormones are long-distanced signaling molecules that must be transported via the circulatory system from their production site to their target cells and may be involved in the radiation induced bystander response for transmitting the signals to the far distanced organs. Another agent can be Ca2^+ ^signaling molecule implicated in the BE [40].

## Implications of DNA damage

BE can be mediated through an increase in chromosomal anomalies, genomic instability, changes in proteins expression, mutations and further by malignant transformation. However, the mechanisms of BE are not yet clear and little is known about the type of DNA damage of the bystander cells, its contributories to tumorigenesis and how this damage can be repaired to design a novel therapeutic approaches to cancer treatment. Ionizing radiation induced DNA damage in a single cell [41] is central to major biological process, such as cancer. However, this classical paradigm has been modified and shown that irradiated cells can also elicit increased level of mutations and chromosomal aberrations in neighboring cells that have never been exposed to radiation. Using high LET radiations (alpha particles), Nagasawa and Little [42] first showed sister chromatid exchange**s **in the non-irradiated cells. BE on the DNA using high LET radiation was further confirmed by Azzam et al [33,43] who found connexin mediated gap junction transfer of signals involved in this process. Later, Daspande et al [44] suggested NADPH mediated mechanisms are also involved. Lyng et al [45] reported, using human keratinocytes when the medium from irradiated cells (0.5 or 5 Gy) was transferred to non-irradiated cells, an increase in calcium fluxes (within 30 s), loss of mitochondrial membrane potential and increase in reactive oxygen species (ROS) (after 6 h of medium transfer). This study suggested that the initiating events in the cascade that leads to apoptosis induction in non-irradiated cells are by signals produced from the irradiated cells. Further, it has been shown that in very low doses (1 cGy-5 Gy) of ^60^Co gamma radiation, increased cell death by apoptosis, necrosis and further reduced cell cloning efficiency in the cells that were never exposed to radiation [46,47].

A delayed genomic instability was observed in the RKO (human colorectal carcinoma cells) after 5Gy of X-rays. Growth medium conditioned by unstable RKO derivatives induced genomic instability suggesting that these cells can secrete factors that produce bystander responses in the unirradiated cells [48]. Konopaka and Wolny [49] tested the bystander effect in human leukemic K562 cells of chronic myeloid leukemia using the medium transfer method. They compared the effects of antioxidant vitamin C and E on the frequency of micronuclei and apoptotic cells in both directly irradiated and also in the bystander cells. Adding vitamin C or E to cell culture reduced the frequency of micronucleated cells in the bystander cells, indicating that these vitamins may reduce the efficacy (cell killing) of radiotherapy. Transforming growth factor β1 (TGF-β1), which plays a major role in radiation induced fibroblast differentiation and glioma cell survival, also has been found to be involved in bystander response signaling [27,50,51].

Using high LET radiations, Lehnert and Goodwin [52] has also been found that alpha particles caused increase in sister chromatid exchange, an indicator of DNA damage in bystander cells. Cells exposed to the high LET radiations showed DNA double strand breaks [53,54], point mutations [55] and multiple DNA double strand breaks in bystander cells [56]. Suzuki et al [57] showed that the altered chromatin organization induced by deletion might be transmitted as DNA damage memory in the following progeny of the bystander cells. Further, Lyng et al [58] reported that microbeam irradiation of keratinocyte (HPU-G) induced an increase in ROS, a decrease in mitochondrial membrane potential, increased expression of Bcl2 and cytochrome c release from the mitochondria into the cytoplasm with an increase in apoptotic cell death. Irradiation of a single human fibroblast with a single 4HE2+ particle demonstrated a 2 to 3 fold increase in micronucleated and apoptotic death in the adjacent non-irradiated cells [36,59]. Belyakov et al [60] studied micronuclei and apoptotic effects in porcine and human urothelial explants and suggested that the bystander-induced damage depends on the proliferation status of the cells when irradiated to 3He2+ ions. Very recently, cells irradiated by low-dose alpha-particles (1-10 cGy) in a mixed Chinese hamster ovary (CHO) cell system showed micronuclei and double strand breaks in the bystander cell population [61].

## Implications of Cell proliferation

**The most commonly reported BE after exposure to radiation is a decrease in cell survival**, an effect similar to that caused by direct irradiation. However, a very few recent studies reported increases in bystander cell proliferation compared with their directly irradiated counterparts. Increases in bystander cell proliferation may show more adverse effects than the decrease in cell proliferation. Iyer et al [50] have demonstrated that when non-irradiated rat liver epithelial cells (WB-F344) were co-cultured with γ-rays irradiated (0.5 to 20 Gy) cells, proliferation was more rapid in the co-cultured cells compared to the control cell populations. However, when both non-irradiated and irradiated cells were seeded sparsely in such a way to avoid contact with each other, no change in the proliferation rate was observed. Conversely, increased proliferation rate in bystander cells was observed when both non-irradiated and irradiated cells were seeded in high density [62]. The authors have concluded that a direct cell-to-cell contact is much more important for increased proliferation observed in bystander cells than either gap junctions or soluble factors. Mothersill et al [63] also showed an increase in clonogenic survival when medium was irradiated (0.5 Gy, γ-rays) and transferred from the mismatch repair deficient (Raji 10) cells to the mismatch repair proficient (Raji TK^-^) cells. Furthermore, Baskar et al [23] reported that when using low (gamma) or high (alpha) LET radiations, an increase in bystander cell survival (proliferation) occurs. However, the magnitude of stimulation in bystander normal (GM637H) fibroblast cells was different when another normal human lung fibroblast (MRC-5) and immortalized ataxia telangiectasia mutated (ATM) defective (GM5849C) fibroblasts were used for low and high LET radiations. The novel finding of this study was that at the same cellular conditions, both low and high LET radiation increased the clonogenic potential (cell proliferation capacity) of recipient cells. Medium from the ATM defective (GM5849C) cells after γ-irradiation showed less stimulating effect than medium from normal (MRC-5) cells. However, after α- irradiation an inverse effect was reported. This may be due to different signals being released in the medium by low and high LET radiations [64].

Interestingly in the bystander cells, increased clonogenic stimulation can be alleviated by dilution of the irradiated conditioned medium [23,65]. It thus becomes clear that the sources of stimulating properties in the culture medium are soluble factors that have been released from the irradiated cells. In a recent study Han et al [60] reported an increase in bystander cell growth when co-cultured with cells irradiated by low-dose α-particles (1-10 cGy). Subsequently, further studies indicated that nitric oxide (NO) and transforming growth factor -1 (TGF-1) played a role in this increase in cell proliferation [23,61]. The increased nature of cell proliferation with the decrease in cell division time is highly suggestive that the bystander rapid cell proliferation might be carcinogenic.

## Molecular signals involved in the BE

The response of cells to ionizing radiation is complex, involving the activation of a cascade of multiple signal transduction pathways. Cellular responses to physiological and environmental stimuli are mediated by specific signaling cascade that can affect cellular growth, differentiation and cell survival. Activation of protein kinase-C (PKC) family is one of the important and earliest events in a cellular cascade leading to a variety of cellular responses such as cell proliferation and differentiation. It is also an important regulator of radiation-induced apoptosis [4,66,67]. In a recent study, ICM collected from normal human lung fibroblasts (MRC-5) 1 h after 1 Gy, or 10 Gy of γ-irradiation, bystander cells showed an increase in intracellular distribution of PKC isoforms (PKC-βII, PKC-α/β, PKC-θ) in total and subcellular (cytosolic and nuclear) fractions [4]. In an earlier study it has been reported that the medium collected from the cells exposed to either low or high LET radiations increased the bystander cell survival [23]. These studies indicate that the molecules released in the ICM, which further activates the PKC may act as a growth stimulatory effect to the bystander cells. The specific sub-cellular activation of PKC isoforms in bystander cells, demonstrated for the first time by Baskar et al [4], may help to identify the effect of therapeutically used radiation exposure for tumor destructions along with its implications for adjacent non-irradiated cells and organs.

However, the nature of the PKC isoforms signaling response that determines cell survival or death is far from completely understood. Further, Hu et al [66] reported PKCε function both as an anti and pro-apoptotic protein and it is the only PKC isozyme implicated in oncogenesis via radiation induced bystander effects. In this respect, the elevated expression of PKC isoforms PKC-βII, PKC-α/β, PKC-θ and PKCε induced in the bystander cells deserves special attention [5,66]. Interestingly, PKC promotes cell survival in response to ionizing radiation in a variety of experimental models including human carcinoma, glioblastoma, and transformed mouse embryo fibroblast cell lines [68]. Inhibition of PKC leads to increased sensitivity of cells to ionizing radiation and suggest its importance in cellular response to radiation [69]. However, understanding the biological functions of individual PKC isoforms and the cellular pathways in which they participate in radiation induced bystander effect remains mostly unknown.

Recently, Burdak-Rothkamm et al [70] demonstrated a decrease in clonogenic survival in ATM/ATR/DNA-PK proficient non-irradiated bystander cells, but this effect was completely abrogated in ATR and ATM but not DNA-PK deficient bystander cells. This, indicate that the ATM activation in bystander cells is dependent on ATR function. Furthermore, bystander cells over express Cox-2 gene [71,72], a downstream target of mitogen-activated protein kinase (MAPK) pathways involved in radiation responses and linked to bystander processes [58]. It is likely that some common initiating or intermediate steps are involved in bystander cellular activation of PKC and Cox-2 in response to radiation. Activation of PKC as a negative regulator of radiation-induced apoptosis might act as a positive regulator for cell proliferation. While the radiation induced mechanisms are not fully established, it can be postulated that the intercellular signaling molecules originating from directly irradiated cells could play a major role in transferring the damage signals to bystander cells.

## Radiation-induced bystander effects in vivo (animal studies)

Recently, research has been focused on bystander effects using animal models.

It is known for several years that the direct radiation exposure effects are sex-specific [73,74]. Recently, Koturbash et al [75] reported different levels of DNA damage in bystander spleen tissue between male and female mice demonstrating for the first time that the males are more sensitive. Interestingly, significant sex differences in the levels of methylation in spleen tissue were eliminated when the mice were subjected to gonadoectomy. Although this study did not find significant sex differences in the levels of death in bystander cells of the spleen (with a non-significant increase in apoptosis in females), it suggested a role of sex hormones interrelationship between bystander effects and secondary radiation-induced malignancies in males and females. Further, Koturbash et al [76,77] reported an existence of somatic bystander effects in vivo using rodent skin and spleen models, whereby one part of the animal body was exposed to radiation while a medical-grade shield protected another part. X-ray exposure of one side of the animal body caused profound epigenetic changes (DNA methylation, histone modification and RNA-associated silencing) in the unexposed bystander parts of the animal.

It was noted that the DNA double-strand breaks and apoptotic cell death were induced in bystander mouse cerebellum. Accompanying these genetic events, bystander-related tumor induction in the cerebellum of radiosensitive Patched-1 (Ptch1) heterozygous mice after x-ray exposure of the remainder of body, further suggested a mechanism mediated by gap-junctional intercellular communication for transmission of bystander damage to shielded cerebellum. These findings are the first to demonstrate that the bystander radiation responses can initiate tumorigenesis in unexposed tissues in vivo [78]. Lorimore et al [79] showed that macrophages obtained from the bone marrow of irradiated CBA/ca mice induced chromosomal instability as assessed by nonclonal cytogenetic aberrations in the clonal descendants of non-irradiated stem cells. However, similar bystander effects are not found in C57BL/6 mice. This study showed a genotype dependent higher incidence of chromosomal instability as a radiation-induced bystander effect in mice. Further Coates et al [80] reported CBA/ca mice are more sensitive to bone marrow macrophage damage than the C57BL/6 mice. Ilnytskyy et al [81] reported that in mice ionizing radiation induced distinct DNA methylation changes in bystander spleen and skin of animals subjected to single-dose (acute) or fractionated whole-body or cranial (head) exposure to 0.5 Gy of X-rays. Acute radiation exposure resulted in a significant loss of global DNA methylation in the exposed and bystander spleen at 6, 96 h and 14 days after irradiation. However, fractionated irradiation led to hypomethylation in bystander spleen at 6 h after whole body irradiation and 6, 96 h and 14 days after cranial irradiation. Contrarily, changes were seen only 6 h after acute whole body and cranial head irradiation. This study showed that the ionizing radiation induced epigenetic bystander effects triggered by both acute and fractionated exposure can occur in the same organism and are very distinct in different bystander organs. Interestingly these data suggest that the observed in vivo results on the radiation induced genomic instability on bystander responses would be of relevance for human health. Clearly, more animal studies will give a concrete evidence of this existing radiation induced bystander phenomenon.

## Conclusions

Radiation induced bystander effect increases the probability/extent of cellular response to radiation and therefore has important implications for cancer risk assessment following low and high doses of low or high LET radiations, and for the possible formation of secondary cancers after radiation exposure. During the past decade there have been major advances in our understanding of the fundamental mechanisms and biological significance of BE. The situation is important among non-elderly adults, whom the long- term risks of radiation exposure induced BE are more relevant. Particularly, children are inherently more radiosensitive, and further children have more remaining years of life during which a late radiation induced bystander cell could develop cancer. However, the general dogma that only direct radiation causes the genomic instability is challenged by the existence of the recent findings of the BE. Limiting the radiation induced genomic damage of normal cells, which may further lead to cancer formation is key in the field of radiation protection. There is no longer any single normal cell showing mutations or being at risk from the radiation. Instead, the risk can also been carried by bystander cells which may be located adjacent or far from the directly irradiated cells/organs. Furthermore, radiation induced bystander cells are at risk for late genomic instability, which is associated with many cancers. The major challenge in the field is to understand the various molecular mechanisms involved in non-targeted effects and therefore counteract those factors, preventing the signaling and thereafter carcinogenesis process. Most encouragingly, the identification of critical molecules that act as the main 'switches' for sending the signals from the irradiated cells/tissues to the non-irradiated cells/tissues should provide a rationale for the development of new therapeutic strategies.

## Conflict of interests statement

The authors declare that they have no competing interests.

## References

[B1] Hall EJ Radiation, the two-edged sword: cancer risks at high and low doses Cancer J 2000 6 343 350 11131480

[B2] Little JB Genomic instability and bystander effects: a historical perspective Oncogene 2003 22 6978 6987 10.1038/sj.onc.1206988 14557801

[B3] United Nations Scientific Committee on the Effects of Atomic Radiation (UNSCEAR) report Effects of ionizing radiation 2006

[B4] Baskar R Balajee AS Geard CR Hande MP Isoform-specific activation of protein kinase c in irradiated human fibroblasts and their bystander cells Int J Biochem Cell Biol 2008 40 125 134 10.1016/j.biocel.2007.07.002 17709275

[B5] Baskar R Hande MP A comparative study of protein kinase c activation in gamma-irradiated proliferating and confluent human lung fibroblast cells J Radiat Res 2009 50 415 423 10.1269/jrr.08125 19602851

[B6] Little MP Radiation: a dose of the bomb Nature 2003 424 495 496 10.1038/424495a 12891335

[B7] Widener A Radiation increases risk of second primary tumors for childhood survivors J Natl Cancer Inst 2006 98 1507

[B8] Barcellos-Hoff MH Park C Wright EG Radiation and the microenvironment tumorigenesis and therapy Nature Rev Cancer 2005 5 867 875 10.1038/nrc1735 16327765

[B9] Fontenot JD Lee AK Newhauser WD Risk of secondary malignant neoplasms from proton therapy and intensity-modulated x-ray therapy for early-stage prostate cancer Int J Radiat Oncol Biol Phys 2009 74 616 622 19427561 10.1016/j.ijrobp.2009.01.001 PMC4120808

[B10] Hall EJ Cancer caused by x-rays-a random event? Lancet Oncol 2007 8 369 370 10.1016/S1470-2045(07)70113-4 17466892

[B11] Hall EJ The impact of protons on the incidence of second malignancies in radiotherapy Tech Cancer Res Treat 2007 6 31 34 10.1177/15330346070060S405 17668949

[B12] Hall EJ Brenner DJ Cancer risks from diagnostic radiology Br J Radiol 2008 81 362 378 10.1259/bjr/01948454 18440940

[B13] Newhauser WD Fontenot JD Mahajan A Kornguth D Stovall M Zheng Y Taddei PJ Zheng Y Taddei P The risk of developing a second cancer after receiving craniospinal proton irradiation Phys Med Biol 2009 54 2277 2229 10.1088/0031-9155/54/8/002 19305036 PMC4144016

[B14] Taddei PJ Mirkovic D Fontenot JD Giebeler A Zheng Y Kornguth D Mohan R Newhauser WD Stray radiation dose and second cancer risk for a pediatric patient receiving craniospinal irradiation with proton beams Phys Med Biol 2009 54 2259 2275 10.1088/0031-9155/54/8/001 19305045 PMC4142507

[B15] Brenner DJ Elliston CD Hall EJ Berdon WE Estimated risks of radiation-induced fatal cancer from pediatric CT AJR 2001 176 289 296 11159059 10.2214/ajr.176.2.1760289

[B16] Brenner DJ Hall EJ Computed tomography-an increasing source of radiation exposure N Engl J Med 2007 357 2277 2284 10.1056/NEJMra072149 18046031

[B17] Doll R Wakeford R Risk of childhood cancer from fetal irradiation Br J Radiol 1997 70 130 139 9135438 10.1259/bjr.70.830.9135438

[B18] Madhusudan S Middleton MR The emerging role of DNA repair proteins as predictive, prognostic and therapeutic targets in cancer Cancer Treat Rev 2005 31 603 607 10.1016/j.ctrv.2005.09.006 16298073

[B19] Lisby M Rothstein R DNA damage checkpoints and repair centers Curr Opin Cell Biol 2004 16 328 334 10.1016/j.ceb.2004.03.011 15145359

[B20] Lukas J Lukas C Bartek J Mammalian cell cycle checkpoints: signaling pathways and their organization in space and time DNA Repair 2004 3 997 1007 10.1016/j.dnarep.2004.03.006 15279786

[B21] Balajee AS Ponnaiya B Baskar R Geard CR Induction of replication protein A in bystander cells Radiat Res 2004 162 677 686 10.1667/RR3269 15548118

[B22] Ballarini F Biaggi M Ottolenghi A Sapora O Cellular communication and bystander effects: A critical review for modeling low-dose radiation action Mutat Res 2002 50 1 12 10.1016/s0027-5107(02)00010-6 11934432

[B23] Baskar R Balajee AS Geard CR Effects of low and high LET radiations on bystander human lung fibroblast cell survival Int J Biochem and Cell Biol 2007 83 551 559 10.1080/09553000701384499 17613128

[B24] Hall EJ Hei TK Genomic instability and bystander effects induced by high LET radiation Oncogene 2003 22 7034 7042 10.1038/sj.onc.1206900 14557808

[B25] Hamada N Matsumoto H Hara T Kobayahsi Y Intercellular and intracellular signaling pathways mediating ionizing radiation-induced bystander effects J Radiat Res 2007 48 87 95 10.1269/jrr.06084 17327686

[B26] Mothersill C Seymour C Radiation-induced bystander effects: Past history and future directions Radiat Res 2001 155 757 765 10.1667/0033-7587(2001)155[0759:RIBEPH]2.0.CO;2 11352757

[B27] Barcellos-Hoff MH Brooks AL Extracellular signaling through the microenviron -ment: a hypothesis relating carcinogenesis, bystander effects and genomic instability Radiat Res 2001 5 618 627 10.1667/0033-7587(2001)156[0618:ESTTMA]2.0.CO;2 11604083

[B28] Mothersill C Seymour CB Cell-cell contact during gamma irradiation is not required to induce a bystander effect in normal human keratinocytes: evidence for release during irradiation of a signal controlling survival into the medium Radiat Res 1998 3 256 262 10.2307/3579958 9496888

[B29] Azzam EI de Toledo SM Walker AJ Little JB High and low fluences of α-particles induce a G_1 _checkpoint in human diploid fibroblasts Cancer Res 2000 60 2623 2631 10825133

[B30] Harada K Nonaka T Hamada H Sakurai H Hasegawa M Funayama T Kakizaki T Kobayashi Y Nakano T Heavy-ion-induced bystander killing of human lung cancer cells: Role of gap junctional intercellular communication Cancer Sci 2009 100 684 688 10.1111/j.1349-7006.2009.01093.x 19469013 PMC11159273

[B31] Mesnil M Piccoli C Tiraby G Willecke K Yamasaki H Bystander killing of cancer cells by herpes simplex virus thymidine kinase gene is mediated by connexins Proc Natl Acad Sci USA 1996 5 1831 1835 10.1073/pnas.93.5.1831 PMC39867 8700844

[B32] Tucker SL Zhang M Dong L Mohan R Kuban D Thames HD Cluster model analysis of late rectal bleeding after IMRT of prostate: A case-control study Int J Radiat Oncol Biol Phys 2006 64 1255 1264 16504763 10.1016/j.ijrobp.2005.10.029

[B33] Azzam EI de Toledo SM Gooding T Little JB Intercellular communication is involved in the bystander regulation of gene expression in human cells exposed to very low fluences of alpha particles Radiat Res 1998 150 497 504 10.2307/3579865 9806590

[B34] Liu Z Mothersill CE McNeill FE Lyng FM Byun SH Seymour CB Prestwich WV A dose threshold for a medium transfer bystander effect for a human skin cell line Radiat Res 2006 166 19 23 10.1667/RR3580.1 16808607

[B35] Mothersill C Seymour CB Medium from irradiated human epithelial cells but not human fibroblasts reduces the clonogenic survival of unirradiated cells Int J Radiat Biol 1997 71 421 427 10.1080/095530097144030 9154145

[B36] Prise KM Belyakov OV Folkard M Michael BD Studies of bystander effects in human fibroblasts using a charged particle microbeam Int J Radiat Biol 1998 74 793 798 10.1080/095530098141087 9881726

[B37] Ponnaiya B Jenkins-Baker G Brenner DJ Hall EJ Randers-Pehrson G Geard CR Biological responses in known bystander cells relative to known microbeam-irradiated cells Radiat Res 2004 162 426 432 10.1667/RR3236 15447040

[B38] Shao C Furusawa Y Aoki M Matsumoto M Ando K Nitric oxide mediated bystander effect induced by heavy-ions in human salivary gland tumour cells Int J Radiat Biol 2001 78 837 844 10.1080/09553000210149786 12428924

[B39] Shao C Stewart V Folkard M Michael BD Prise KM Nitric oxide-mediated signaling in the bystandard response of individually targeted glioma cells Cancer Res 2003 63 8437 8442 14679007

[B40] Sammak PJ Hinman LE Tran PO Sjaastad MD Machen TE How do injured cells communicate with the surviving cell monolayer? J Cell Sci 1997 110 465 475 9067598 10.1242/jcs.110.4.465

[B41] Rothkamm K Löbrich M Evidence for a lack of DNA double-strand break repair in human cells exposed to very low x-ray doses Proc Natl Acad Sci USA 2003 100 5057 5062 10.1073/pnas.0830918100 12679524 PMC154297

[B42] Nagasawa H Little JB Induction of sister chromatid exchanges by extremely low doses of alpha-particles Cancer Res 1992 52 6394 6396 1423287

[B43] Azzam EI de Toledo SM Little JB Direct evidence for the participation of gap junction-mediated intercellular communication in the transmission of damage signals from α-particle irradiated to nonirradiated cells Proc Natl Acad Sci USA 2001 98 473 478 10.1073/pnas.011417098 11149936 PMC14611

[B44] Deshpande A Goodwin EH Bailey SM Marrone BL Lehnert BE Alpha-particle-induced sister chromatid exchange in normal human lung fibroblasts: evidence for an extranuclear target Radiat Res 1996 145 260 267 10.2307/3578980 8927692

[B45] Lyng FM Seymour CB Mothersill C Early events in the apoptotic cascade initiated in cells treated with medium from the progeny of irradiated cells Radiat Prot Dosim 2002 99 169 172 10.1093/oxfordjournals.rpd.a006753 12194275

[B46] Mothersill C Stamato TD Perez ML Cummins R Mooney R Seymour CB Involvement of energy metabolism in the production of bystander effects by radiation Br J Cancer 2000 82 1740 1746 10.1054/bjoc.2000.1109 10817512 PMC2374513

[B47] Mothersill C Seymour CB Bystander and delayed effects after fractionated radiation exposure Radiat Res 2002 158 626 633 10.1667/0033-7587(2002)158[0626:BADEAF]2.0.CO;2 12385640

[B48] Huang L Kim PM Nickoloff JA Morgan WF Targeted and nontargeted effects of low-dose ionizing radiation on delayed genomic instability in human cells Cancer Res 2007 67 1099 1104 10.1158/0008-5472.CAN-06-3697 17283143

[B49] Konopacka M Rzeszowska-Wolny RJ The bystander effect-induced formation of micronucleated cells is inhibited by antioxidants, but the parallel induction of apoptosis and loss of viability are not affected Mutat Res 2006 593 32 38 16040062 10.1016/j.mrfmmm.2005.06.017

[B50] Iyer R Lehnert BE Svensson R Factors underlying the cell growth-related bystander responses to alpha particles Cancer Res 2000 60 1290 1298 10728689

[B51] Gow MD Seymour CB Ryan LA Mothersill CE Induction of Bystander Response in Human Glioma Cells using High-Energy Electrons: A Role for TGF-β Radiat Res 2010 173 769 778 10.1667/RR1895.1 20518656

[B52] Lehnert BE Goodwin EH A new mechanism for DNA alterations induced by alpha particles such as those emitted by radon and radon progeny Environ Health Perspect 1997 105 1095 1101 10.2307/3433515 9400706 PMC1470136

[B53] Nagasawa H Little JB Bystander effect for chromosomal aberrations induced in wild-type and repair deficient CHO cells by low fluencies of alpha particle Mutat Res 2002 508 121 129 12379467 10.1016/s0027-5107(02)00193-8

[B54] Nagasawa H Cremesti A Kolesnick R Fuks Z Little JB Involvement of membrane signaling in the bystander effect in irradiated cells Cancer Res 2002 62 2531 2534 11980645

[B55] Zhou H Randers-Pehrson G Waldren CA Vannais D Hall EJ Hei TK Induction of a bystander mutagenic effect of alpha particles in mammalian cells Proc Natl Acad Sci USA 2000 97 2099 2104 10.1073/pnas.030420797 10681418 PMC15760

[B56] Morgan WF Non-targeted and delayed effects of exposure to ionizing radiation: II. Radiation-induced genomic instability and bystander effects in vivo, clastogenic factors and transgenerational effects Radiat Res 2003 159 581 596 10.1667/0033-7587(2003)159[0581:NADEOE]2.0.CO;2 12710869

[B57] Suzuki M Zhou H Geard CR Hei TK Effect of medium on chromatin damage in bystander mammalian cells Radiat Res 2004 162 264 269 10.1667/RR3226 15332998

[B58] Lyng FM Maguire P McClean B Seymour C Mothersill C The involvement of calcium and MAP kinase signaling pathways in the production of radiation-induced bystander effects Radiat Res 2006 165 400 409 10.1667/RR3527.1 16579652

[B59] Belyakov OV Malcolmson AM Folkard M Prise KM Michael BD Direct evidence for a bystander effect of ionizing radiation in primary human fibroblasts Br J Cancer 2001 84 674 679 10.1054/bjoc.2000.1665 11237389 PMC2363796

[B60] Belyakov OV Folkard M Mothersill C Prise KM Michael BD A proliferation-dependent bystander effect in primary porcine and human urothelial explants in response to targeted irradiation Br J Cancer 2003 88 767 774 10.1038/sj.bjc.6600804 12618888 PMC2376355

[B61] Han W Chen S Yu KN Wu L Nitric oxide mediated DNA double strand breaks induced in proliferating bystander cells after particle irradiation Mutat Res 2010 684 81 89 20026341 10.1016/j.mrfmmm.2009.12.004

[B62] Gerashchenko BI Howell RW Cell proximity is a prerequisite for the proliferative response of bystander cells co-cultured with cells irradiated with γ-rays Cytometry 2003 56 71 80 10.1002/cyto.a.10092 14608634

[B63] Mothersill C Seymour RJ Seymour CB Increased radiosensitivity in cells of two human cell lines treated with bystander medium from irradiated repair deficient cells Radiat Res 2006 165 26 34 10.1667/RR3488.1 16392959

[B64] Wright EG Coates PJ Untargeted effects of ionizing radiation: implications for radiation pathology Mutat Res 2006 597 119 132 16438994 10.1016/j.mrfmmm.2005.03.035

[B65] Ryan LA Smith RW Seymour CB Mothersill CE Dilution of Irradiated Cell Conditioned Medium and the Bystander Effect Radiat Res 2008 169 188 196 10.1667/RR1141.1 18220470

[B66] Hu B Shen B Su Y Geard CR Balajee AS Protein kinase C epsilon is involved in ionizing radiation induced bystander response in human cells Int J Biochem Cell Biol 2009 41 2413 2421 10.1016/j.biocel.2009.06.012 19577658 PMC2784166

[B67] Yoshida K Nuclear trafficking of pro-apoptotic kinases in response to DNA damage Trends Mol Med 2008 14 305 313 10.1016/j.molmed.2008.05.003 18539531

[B68] McCraken MA Miraglia LJ Mckay RA Strobl JS Protein kinase c is a prosurvival factor in human breast tumor cell lines Mol Cancer Ther 2003 2 273 281 12657722

[B69] Choi EK Rhee YH Park HJ Ahn SD Shin KH Park KK Effect of protein kinase C inhibitor (PKCI) on radiation sensitivity and c-fos transcription Int J Radiat Oncol Biol Phys 2001 49 397 405 10.1016/S0360-3016(00)01485-1 11173133

[B70] Burdak-Rothkamm S Rothkamm K Prise KM ATM Acts Downstream of ATR in the DNA Damage Response Signaling of Bystander Cells Cancer Res 2008 68 7059 7065 10.1158/0008-5472.CAN-08-0545 18757420 PMC2528059

[B71] Hei TK Cyclooxygenase-2 as a signaling molecule in radiation-induced bystander effect Mol Carcinogen 2006 45 455 460 10.1002/mc.20219 16637062

[B72] Zhou H Ivanov VN Gillespie J Geard CR Amudson SA Brenner DJ Yu Z Lieberman HB Hei TK Mechanism of radiation-induced bystander effect: role of the cyclooxygenase-2 signaling pathway Proc Natl Acad Sci USA 2005 102 14641 14646 10.1073/pnas.0505473102 16203985 PMC1253564

[B73] Besplug J Burke P Ponton A Filkowski J Titov V Kovalchuk I Kovalchuk O Sex and tissue-specific differences in low-dose radiation-induced oncogenic signaling Int J Radiat Biol 2005 81 157 168 10.1080/09553000500103512 16019925

[B74] Guerquin MJ Duquenne C Coffigny H Rouiller-Fabre V Lambrot R Bakalska M Frydman R Habert R Livera G Sex-specific differences in fetal germ cell apoptosis induced by ionizing radiation Hum Reprod 2009 24 670 678 10.1093/humrep/den410 19088112

[B75] Koturbash I Loree J Kutanzi K Koganow C Pogribny I Kovalchuk O In vivo bystander effect: cranial X-irradiation leads to elevated DNA damage and altered cellular proliferation and apoptosis in shielded spleen Int J Radiat Oncol Biol Phys 2008 70 554 562 18207032 10.1016/j.ijrobp.2007.09.039

[B76] Koturbash I Rugo RE Hendricks CA Loree J Thibault B Kutanzi K Pogribny I Yanch JC Engelward BP Kovalchuk O Irradiation induces DNA damage and modulates epigenetic effectors in distant bystander tissue in vivo Oncogene 2006 25 4267 4275 10.1038/sj.onc.1209467 16532033

[B77] Koturbash I Boyko A Rodriguez-Juarez R McDonald RJ Tryndyak VP Kovalchuk I Pogribny IP Kovalchuk O Role of epigenetic effectors in maintenance of the long-term persistent bystander effect in spleen in vivo Carcinogenesis 2007 28 1831 1838 10.1093/carcin/bgm053 17347136

[B78] Mancuso M Pasquali E Leonardi S Tanori M Rebessi S Di Majo V Pazzaglia S Toni MP Pimpinella M Covelli V Saran A Oncogenic bystander radiation effects in Patched heterozygous mouse cerebellum Proc Natl Acad Sci USA 2008 105 12445 12450 10.1073/pnas.0804186105 18711141 PMC2517601

[B79] Lorimore SA Chrystal JA Robinson JI Coates PJ Wright EG Chromosomal instability in unirradiated hemaopoietic cells induced by macrophages exposed in vivo to ionizing radiation Cancer Res 2008 68 8122 8126 10.1158/0008-5472.CAN-08-0698 18829571

[B80] Coates PJ Rundle JK Lorimore SA Wright EG Indirect Macrophage Responses to Ionizing Radiation: Implications for Genotype-Dependent Bystander Signaling Cancer Res 2008 68 450 456 10.1158/0008-5472.CAN-07-3050 18199539

[B81] Ilnytskyy Y Koturbash I Kovalchuk O Radiation-induced bystander effects in vivo are epigenetically regulated in a tissue-specific manner Environ Mol Mutagen 2009 50 105 113 10.1002/em.20440 19107897

